# The Medical Society of the County of Erie

**Published:** 1905-04

**Authors:** Myrtle H. Massey


### SOCIETY PROCEEDINGS.

### The Medical Society of the County of Erie.

*Special Meeting, February 27, 1905.*

REPORTED BY MYRTLE H. MASSEY, M. D., Secretary pro tem.

THE vice-president, Dr. A. H. Briggs, took the chair at 4.15 p. m., and in appropriate terms announced the death of former president, Dr. E. C. W. O'Brien, and asked the society to take such action as would befit the occasion. Dr. William Warren Potter said that it would seem proper to follow the precedents of the society, and moved that the chair appoint a committee of five to prepare a memorial of Dr. O'Brien. This was carried and the vice-president appointed as such committee, Drs. William Warren Potter, Henry R. Hopkins, DeLancey Rochester, S. S. Green, and J. M. O'Neill. The committee subsequently reported the following:

### IN MEMORIAM, EDWARD C. W. O'BRIEN, M. D., 1843-1905.

Edward Charles White O'Brien was born at Quebec, February 4, 1843, and died at Buffalo, February 26, 1905, aged 62 years. He received his preliminary education in the city of his birth principally from the Christian brothers and at private schools. At an early age he was compelled to earn his own living and after a few years hard work, which developed strength of character and a resourceful mentality, he decided to enter the profession of medicine. He came to Buffalo in 1859 and, after a few years, entered the Medical Department of the University of Buffalo from which he was graduated in 1867.

Dr. O'Brien began the practice of medicine almost immediately after graduation and continued his professional work until his last illness. He soon acquired the confidence of his colleagues and was a favorite with many of the older physicians. Dr. James P. White often sought his services as an assistant at operations, especially at distances more or less remote from the city. Besides being useful at the operating table he was an agreeable companion, and Drs. White and O'Brien always created a favorable impression during their professional excursions into the country.

Early in his professional career—namely, in 1872, Dr. O'Brien was appointed health physician of Buffalo, which then was the title of the office now known as health commissioner. He served in this office until 1877. During his incumbency several important events occurred, the most notable of which, perhaps, was an epidemic of smallpox which raged throughout the country, and from which Buffalo suffered to the extent of about 1,900 cases. A smallpox hospital was established during the epidemic which Dr. O'Brien regularly visited twice a day, often late at night, in the discharge of his unpleasant duty. He made large use of bovine virus and is credited with being the first to use it here in any considerable way, meeting opposition thereto from the public as well as from some physicians.

Dr. O'Brien's service as health officer gave him prominence not only at home but throughout the state. This, no doubt, at least in a measure, subsequently led to his nomination as health officer of the port of New York, for which he was recommended by a large number of physicians and political friends, many of whom appeared before the senate committee to whom the nomination was referred, and urged his confirmation. He, however, failed of confirmation because of factional differences among republicans which existed at that time.

Dr. O'Brien had held many professional places of honor or trust. For about ten years he was surgeon of the 74th regiment, N. G. N. Y.; for many years chief medical examiner of the Catholic Mutual Benefit Association of the state of New York; attending physician at Saint Vincent's Orphan Asylum; consulting physician at Providence Retreat (hospital for the insane); consulting surgeon to the Riverside Hospital, and surgeon to the Buffalo fire department.

Dr. O'Brien was a member of the American Medical Association; of the Medical Society of the County of Erie (president in 1892); of the Buffalo Academy of Medicine; of the Alumni Association of the University of Buffalo (president, 1885), and a curator of the University of Buffalo, holding the latter place at the time of his death.

Perhaps during Dr. O'Brien's long and successful career nothing has been more accentuated than his faithful and efficient service rendered the fire department. It was his habit to respond to all second alarms turned in, night or day, in order to be present should his services be needed by any of the officers or men of the department. During his last illness anxious inquiries were constantly made by the firemen as to his condition and hope for his recovery was often expressed by the men whom he served so well. All the fire stations displayed flags at half mast upon receiving notification of his death.

Dr. O'Brien was a striking figure of well developed manhood, familiar to a large majority of our inhabitants, who will miss him in his public and private relations to the city and her citizens. He was an upright man, a courteous gentleman, an accomplished physician, and a faithful friend. Dr. O'Brien married Miss Monterey Allis, of New York city, October 8, 1879. His widow without issue survives.

In token of respect to the deceased the following resolutions are offered:

*Resolved,* That the Medical Society of the County of Erie has heard with emotions of sorrow and regret of the death of one of its distinguished members and former presidents, Dr. Edward C. W. O'Brien, and votes to spread the foregoing memorial upon its minutes.

*Resolved,* That a copy thereof, including these resolutions, be transmitted to his widow, with tenderest expressions of sympathy.

[Signed]
WILLIAM WARREN POTTER,
HENRY R. HOPKINS,
DE LANCEY ROCHESTER,
S. S. GREEN,
J. M. O'NEIL.

A recess was then taken on motion of Dr. Henry R. Hopkins, who also moved that the physicians present organise a meeting in the interest of the public health. This being seconded and carried, Dr. Hopkins further moved that Dr. DeLancey Rochester be invited to the chair. Carried. Dr. Myrtle L. Massey, who had been chosen secretary pro tem of the previous meeting, was elected acting secretary.

Dr. Rochester, on taking the chair, stated the object of the meeting under the invitation of the Medical Society of the County of Erie, to be in particular to take action concerning certain bills pending in the state legislature, which were believed to be inimical to the interests of the people of the state of New York.

At the request of the chair, Dr. Hopkins read Senate bill No. 298, introduced by Senator Davis, of Erie, purporting to regulate the practice of osteopathy.

Dr. William Warren Potter called attention to the dangerous character of such legislation. This bill, if passed, would add hundreds of names to the roll of physicians in this state. It was cunningly devised by shrewd men; in one place it restricted the practice of the osteopath, while in another it gave him all the rights of "other physicians." It was difficult to understand why such a bill could be taken seriously by the legislature and especially by the senate. If it should pass it would create a new schism and elevate to the dignity of a profession a system that has no such right. The University of the State of New York,—the department of education,—would be debased by such action and the great educational head of the state would be dragged down to the level of a low order of commercialism. He warned the entire medical profession of the dangers which threatened it and urged it to take such action as would arouse public interest on the subject.

Dr. S. S. Greene said that the bill was perfectly absurd on its face; that the chiropodists and the massage people might as well go to Albany and ask to be chartered as medical societies. He did not believe our legislators would listen to such an offensive proposition, but he recognised the uncertainties that might attend neglect and urged active opposition to the bill.

Dr. F. H. Millener read an editorial from the *Evening News* favoring the bill. This paper has generally been found on the right side of the professional education question, but in this instance had gone to the very extreme of inconsistency.

Dr. William C. Krauss urged that a strong protest be made through a representative delegation to Albany, at the hearing appointed to consider the bill. He urged that the situation be not regarded with triviality, but as a grave condition requiring strong measures to meet and antagonise.

Dr. Lucien Howe also considered it important that a special committee should proceed to Albany and oppose the bill in strong terms at the hearing.

Dr. A. A. Hubbell agreed with the previous speakers and also urged that the committee be instructed to oppose the optometry bill, which has again appeared in evidence at Albany. He thought the committee should be instructed to oppose the bills relating to osteopathy, optometry, kinesipathy, mental science or any other similar disgraceful attempt to lower educational standards or endanger the public health.

Dr. Ernest Wende said that he opposed the osteopathic bill and all similar measures not as a representative of the medical profession, but in behalf of the people. He blamed the public press for the standing it gave to quacks and nostrums, stating that they would have to go out of business were it not for the advertising their goods obtained in the newspapers which, in his judgment, made the latter particeps criminis in the deception of the people.

Dr. Henry R. Hopkins declared the bill exceedingly dangerous because, for one reason, it would license hundreds, perhaps thousands of men now engaged in the criminal violation of the law under the guise of the so-called practice of osteopathy. Under the provisions of the proposed bill none of these men will be required to meet the educational tests provided by the state for "other physicians," borrowing this term, "other physicians," from the language of the bill. Physicians, forsooth! without having studied medicine in all its several branches, not to mention lack of preliminary education. He declared the bill to be contrary to the spirit of our institutions, against progress in professional and all other education and as jeopardising the public health. It turns the clock back to the conditions of the eighteenth century instead of keeping it abreast of the progress everywhere making in this enlightened twentieth century of superior attainments.

Dr. Hopkins then presented the following preamble and resolutions and moved their adoption:

WHEREAS, The state of New York, by a wise use of her sovereign right has, for many years, maintained a severe test of all persons seeking the right to practise medicine among her people; and,

WHEREAS, This state test has been exercised chiefly in determining the candidate's preliminary education, the time spent in gaining his medical equipment, the advantage the candidate had actually taken of these opportunities for general and professional preparation, and as to his good moral character; and,

WHEREAS, The wisdom of this policy on the part of the state of New York has been amply justified in the imitation thereof by more than forty states and territories of America; and,

WHEREAS, The medical profession of the state of New York is justly proud of her standard of professional preparedness and jealous that the same be intelligently maintained; and,

WHEREAS, A bill is now pending at Albany, known as Senate bill No. 298, which we have heard read; and,

WHEREAS, By the provisions of said bill a large number of persons are given the right to practise medicine in this state without test by the state of their preparedness for such great responsibility; therefore be it,

*Resolved,* That we the medical profession of the county of Erie, state of New York, are opposed to Senate bill No. 298, as a step backward in educational standards and as inimical to the public health.

*Resolved,* That we call upon our representatives,—Senators and Members of Assembly,—to oppose with all honorable means Senate bill No. 298.

*Resolved,* That a committee to be named by the chair is hereby instructed to visit Albany, to appear at hearings, and in all proper methods to execute the will of the medical profession of Erie county in opposing Senate bill No. 298.

*Resolved,* That a copy of these resolutions, duly signed and executed, be sent to each of our respresentatives at Albany.

The motion was duly seconded and carried as was also a motion that the chair appoint a committee of five to proceed to Albany and oppose the several bills referred to in the proceedings.

The chair then named the committee as follows: Henry R. Hopkins, Ernest Wende, William C. Krauss, A. H. Briggs, and Edward Clark.

The meeting was attended by twenty-five physicians and adjourned at 5.30 p. m.

